# Psychological mediators of the relations between goal motives, physical activity and well-being: Testing a model of path analysis

**DOI:** 10.1177/13591053251330430

**Published:** 2025-04-15

**Authors:** Katie R. Garstang, Daniele Magistro, Patricia C. Jackman, Simon B. Cooper, Laura C. Healy

**Affiliations:** 1 Sport, Health and Performance Enhacement (SHAPE) Research Group, School of Science and Technology, Nottingham Trent University, UK; 2 Centre for Behavioural Science and Applied Psychology, Institute of Social Sciences, Sheffield Hallam University, UK; 3 School of Psychology, Sport Science and Wellbeing, University of Lincoln, UK

**Keywords:** motivation, exercise, self-efficacy, affect, well-being, goal pursuit

## Abstract

The autonomous and controlled motivations underpinning goal pursuit directly impact physical activity and mental well-being and are important for healthy behavior adherence. Psychological variables can also affect physical activity and mental well-being. This study tested the association between goal motives, psychological variables, physical activity, and mental well-being using structural equation modelling. Adults (*N* = 323; mean age =32.46 ± 13.12 y) completed a cross-sectional survey measuring goal motives, motivation, affective experiences, self-efficacy, physical activity, and mental well-being. Our analysis showed support for the proposed model fit: (χ^2^(6) = 14.16, *p* = .028, RMSEA = .07, CFI = .99, TLI = .97). In contrast to controlled goal motives, autonomous goal motives were positively related to the psychological variables associated with physical activity and mental well-being. Motivation and affective experiences were positively associated with physical activity. Self-efficacy was positively associated with mental well-being. Intricacies of the associations between goal motives, psychological variables, physical activity, and mental well-being are discussed.

Higher levels of sedentary behavior and lower levels of physical activity are associated with increased risks of health issues and mortality (Hechanova et al., 2017). When aiming to increase an individual’s long-term engagement in physical activity, it is also important to consider relationships with mental well-being, defined as a combination of feeling good and functioning effectively (Stewart-Brown et al., 2011). Greater mental well-being can predict repeated, continuous physical activity behaviors (Rector et al., 2019) and is considered fundamental for optimal physical health (Biddle and Mutrie, 2007). When seeking to promote mental wellbeing, health and exercise practitioners are usually encouraged to help individuals set physical activity goals (e.g. Cooper, 2020). Goal setting is a widely used and effective technique for increasing physical activity (Howlett et al., 2019; McEwan et al., 2016). Although goals people set are underpinned by motives (Sheldon and Elliot, 1999), many goal-setting interventions fail to consider the underlying reasons for engaging in specific behaviors, the psychological variables that may influence these, and relationships with mental well-being. Therefore, this study examined how motives underpinning goal pursuit were associated with physical activity and mental well-being.

In the self-concordance model (SCM), Sheldon and Elliot (1999) proposed well-being as the main outcome of goal striving. Within the SCM, two overarching goal motives are proposed: autonomous goal motives (i.e. motives that hold intrinsic value and are of personal interest to the individual); and controlled goal motives (i.e. an individual feels compelled to do something due to internal or external pressures). Both goal motives can be powerful drivers of goal striving, but the long-term impacts of these goal motives can vary. Controlled motives may initially change behaviors yet are unlikely to result in long-term behavior change as the effort invested in goal pursuit can fade over time (Sheldon and Elliot, 1998). Furthermore, controlled motives are negatively related to perceived mental well-being (Briki, 2016; Ng et al., 2012) and unrelated to moderate-intensity physical activity (Standage et al., 2008) and the maintenance of healthy behaviors (Ng et al., 2012).

In contrast, goals pursued with autonomous motives have not only been linked to achievement of desired outcomes, but also to improved well-being (Ntoumanis et al., 2014) and more generally to psychological health (Deci and Ryan, 2008). Furthermore, all forms of autonomous regulation can predict exercise and physical activity participation (Teixeira et al., 2012), highlighting the potential benefits of more self-concordant and autonomous goals for long-term physical activity adherence. While researchers have assessed the direct effects of goal motives on exercise and physical activity (Teixeira et al., 2012), there is limited understanding of how the effects of goal motives on physical activity and mental well-being might be mediated via other psychological variables. Thus, further research is needed to better understand how different psychological variables relate to individuals’ goal motives for physical activity, and the subsequent impacts upon long-term physical activity adherence and overall mental well-being.

One of the most important correlates of physical activity behavior is self-efficacy (Bauman et al., 2012). In the context of physical activity promotion, researchers have found that self-efficacy is positively associated with increased vigorous-intensity physical activity (Sallis et al., 1989), decreased sedentary behavior (Szczuka et al., 2021), and is a strong predictor of exercise behaviors for those in the initial stages of starting to be physically active (McAuley and Blissmer, 2000). In turn, this suggests that self-efficacy could play a vital role in one’s intent and pursuit of long-term physical activity behaviors. Despite suggestions that individuals who pursue more self-concordant goals are more likely to feel more competent and effective (Sheldon and Elliot, 1999), research is needed to empirically examine the relationship between goal motives and self-efficacy in the context of physical activity.

Motivation is another psychological variable that has been shown to be a direct determinant of behavior (Knittle et al., 2018), including prolonged physical activity (≥10 weeks; Wilson and Rogers, 2007). While there are many types of motivation (Ryan and Deci, 2017), it is generally accepted that more self-determined motivation is more likely to lead to behavioral adoption and maintenance (Weman-Josefsson et al., 2015). In terms of physical activity behavior, research shows that autonomous motivation, and more specifically integrated motivation, is particularly influential for promoting physical activity (Sevil et al., 2015). These findings suggest that autonomous forms of motivation are key for physical activity behaviors, but further research is needed to understand how goal motives are related to motivation for physical activity, and the subsequent influence on mental well-being.

Finally, in recent years, there has been increased recognition of the importance of affective experiences in promoting physical activity (Ekkekakis and Zenko, 2016). When individuals experience more pleasure in physical activity, they are more likely to approach this behavior again in future, whereas unpleasant experiences are more likely to lead to avoidance behaviors (Ekkekakis and Brand, 2019). Therefore, it is proposed that if an individual has an unpleasant affective experience with physical activity, this could lessen the likelihood of them engaging in physical activity (Ekkekakis et al., 2021) and thus negatively impact their perceived mental well-being. While evidence continues to accumulate on the relationship between affective experience and physical activity behaviors, further research is needed to examine whether different goal motives for physical activity elicit different affective experiences in physical activity, and how these experiences might subsequently be related to physical activity behaviors and mental well-being.

In this study, we aimed to enhance understanding of the influence goal motives have on psychological variables associated with physical activity (i.e. self-efficacy, motivation, and affect), physical activity, and mental well-being in adults, and examine the relationships between these variables. In addressing this aim, we sought to answer the following questions: (a) are psychological variables associated with increased physical activity and improved mental well-being linked to one’s motives for pursuing physical activity goals?; (b) how do self-efficacy, motivation, and affective exercise experiences link to physical activity and mental well-being?; and (c) how do these psychological variables mediate the relationship between motives for physical activity and both physical activity and mental well-being?

We hypothesized that: (H1) goal motives would be directly associated with self-efficacy, motivation, and affective exercise experiences, and expected autonomous motives to show positive associations, and controlled motives to have negative associations; (H2) self-efficacy, motivation, and affective exercise experiences would be directly, positively associated with physical activity and mental well-being; and (H3) goal motives would be indirectly associated with physical activity and mental well-being via self-efficacy, motivation and affective exercise experiences. Therefore, in our proposed model (Figure 1), we suggested that the motives underpinning physical activity goals would not be directly related to physical activity and well-being; instead, we hypothesized that one’s belief in their ability to achieve the goal, the quality of motivation one has striving for the goal, and their affective exercise experiences should be considered, in addition to goal motives, when examining factors related to physical activity and perceived well-being. The effect of these autonomous and controlled motives on self-efficacy, motivation, and affective exercise experience, in turn, was posited to influence physical activity behaviors and perceived mental well-being. By considering how goal motives might be related to both physical activity and mental well-being together, we sought to develop evidence that could provide a platform to enhance goal-setting interventions in future.

**Figure 1.**
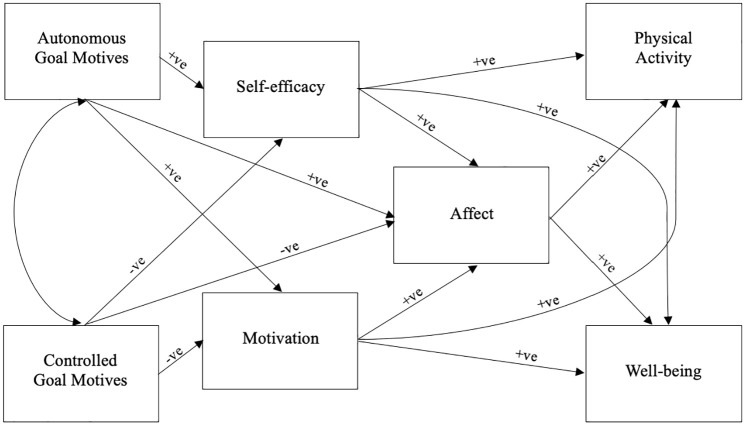
Conceptual model of the associations between goal motives, psychological variables, physical activity and well-being. *Note.*+ve predicted a positive association; −ve predicted a negative association.

## Methods

### Participants

A total of 368 individuals completed an online survey on a single occasion. This sample size was accepted based on the *N*:q ratio, minimum recommendation of 20:1 (participants:parameter), suggestive that a minimum sample of 140 participants was required to perform the analysis (Kline, 2016; Kyriazos, 2018). Individuals were all living in the United Kingdom at the time of completing the survey. Responses were recorded between June 2021 and February 2023.

### Procedure

After receiving ethical approval (ID: 20/21-97), a JISC online survey was distributed through social media, posters, and word of mouth. After reading the information sheet and providing informed consent, respondents were asked to complete sets of questions in the following order: demographics, current physical activity levels, mental well-being, affective exercise experiences, motivation, self-efficacy, and goal motives. The average duration to complete the survey was 30.14 minutes, and respondents did not receive any compensation for participating.

### Measures

#### Goal motives

To measure goal motives, we utilized a 4-item questionnaire that has been used in prior research (Sheldon and Elliot, 1999). Participants were asked to identify a physical activity goal (e.g. “to stay healthy and fit” or “to feel mentally sharp”) and to rate the extent to which the four items represented their motives for goal pursuit on a 7-point Likert scale ranging from 1 (*Not at all*) to 7 (*Very much so*). The four items were divided into two subscales, representing the two overarching motives: autonomous goal motives (“*Because you personally believe it’s an important goal to have*” and “*Because of the fun and enjoyment the goal provides you*”) and controlled goal motives (“*Because someone else wants you to*” “*Because you would feel ashamed, guilty, or anxious if you didn’t*”). Autonomous and controlled motives were scored by taking an average of the two responses relating to that subscale; the reliability of the subscales were very good (ρ = 0.79) and fair (ρ = 0.36) respectively.

#### Affective Exercise Experiences (AFFEXX) questionnaire

Affective exercise experiences were measured using the AFFEXX questionnaire (Ekkekakis et al., 2021). For the purpose of this study, the single scale of “antipathy-attraction” was used to represent affective experiences and one’s desire to complete physical activity as it is ultimately influenced collectively by the antecedent and core variables of affective experiences that are stated in the measure. The scale is comprised of five items, where questions are phrased as pairs of opposites on a 7-point scale (e.g., *“Exercise is an uninviting activity” = 1 versus “Exercise is a tempting activity” = 7*). Higher scores corresponded with attraction, and lower scores corresponded to antipathy. These subscales were previously reported to correlate with self-reported moderate and vigorous physical activity and have demonstrated very good internal consistency scores (α = 0.92; Ekkekakis et al., 2022). In the current study, internal consistency of the antipathy-attraction subscale was very good (α = 0.88).

#### Behavioral Regulation in Exercise Questionnaire 3 (BREQ-3)

Motivation regulations were measured using the BREQ-3 (Markland and Tobin, 2004; Wilson et al., 2006). The BREQ-3 is a multidimensional measure based on SDT literature offering scores for six subscales (“amotivation,” “external regulation,” “introjected regulation,” “identified regulation,” “integrated regulation,” and “intrinsic regulation”) and a relative autonomy index (RAI) of self-determination. Each of the 24 items was scored on a 5-point Likert scale ranging from 0 to 4 (*0 = Not true for me; 1, 2 = Sometime true for me; 3, 4 = Very true for me*). An average score is calculated for each subscale, and then multiplied by its predisposed weighting, before summing the total weighted scores to provide a RAI score. The higher the score, the greater one’s autonomous motivation. The RAI score was used due to its practicality and ability to predict outcomes (Ryan and Deci, 2017). Various versions of the BREQ scale are consistently used in exercise contexts (Teixeira et al., 2012). The BREQ-3 captures a broader scope of subscales than the previous versions of the scale that compromise an overall scale of motivation and has displayed good internal consistency in adult populations (.66 ≤ α ≤ .75; Vancampfort et al., 2018). In the current study, internal consistency values for the BREQ-3 subscales were very good (.82 ≤ α ≤ .89).

#### Generalized Self-Efficacy Scale (GSE)

Self-efficacy was measured using the GSE (Schwarzer et al., 1995), which contained 10 items (e.g. “*I can always manage to solve difficult problems if I try hard enough*”) scored on a 4-point Likert scale (*1 = Not at all true; 2 = Hardly true; 3 = Moderately true; 4 = Exactly true)*. Self-efficacy was determined by a sum of all items; the higher the score, the higher an individual’s self-efficacy. The GSES has previously displayed very good internal consistency (α = .86; Sherer et al., 1982). In the current study, internal consistency of the GSE was very good (α = .88).

#### Short Warwick-Edinburgh Mental Wellbeing Scale (SWEMWBS)

Perceived mental well-being was captured using the SWEMWBS (Tennant et al., 2007). The SWEMWBS is made up of seven statements about the respondents’ feelings and thoughts over the past 2 weeks. Permission for use of the measure was sought prior to data collection. Respondents reported their answers on a 5-point Likert scale (*1 = None of the time; 2 = Rarely; 3 = Some of the time; 4 = Often; and 5 = All of the time*). The sum of the items is then scored and converted, with higher scores indicative of higher positive mental well-being. The SWEMWBS was selected due to its validity for use with the general population (Ng Fat et al., 2017) and its very good internal consistency (α = .89; Stewart-Brown et al., 2011). In the current study, internal consistency of the SWEMWBS was very good (α = .84).

#### International Physical Activity Questionnaire - short form (IPAQ-short form)

Moderate-to-vigorous physical activity (MVPA) was captured using the IPAQ-short form (Craig et al., 2017). The IPAQ-short form was selected as total moderate and total vigorous activity time is scored in isolation to other types of activity and it has been shown to have a very good internal consistency (α = .80; Craig et al., 2017). Total minutes of moderate and vigorous activity was scored separately and summed to provide 1 minutes of MVPA score.

### Data analysis

SPSS Version 29 was used to screen the data for univariate and multivariate outliers and to produce descriptive statistics. Correlations were performed between all variables. To assess the co-variances between goal motives, the associated psychological variables, physical activity and well-being, structural equation model path analysis was performed using Amos Version 26 software (Arbuckle, 2019). Associations were characterized as follows: small, β ≤ 0.29; moderate: 0.30 ≤ β ≤ 0.49; and large β ≥ 0.50 (Cohen, 2013; Fey et al., 2023). Absolute fit indices were used to determine the best model fit for the data. Hu and Bentler (1999) determined a model to be of good fit if chi-square (χ^2^) was found to be non-significant, the absolute fit measure root mean squared error of approximation (RMSEA; Steiger, 1990) value was below .06, and relative fit measures of the Comparative Fit Index (CFI; Bentler, 1990) and the Tucker-Lewis Index (TLI; Tucker and Lewis, 1973) were ≥ .95 (Hu and Bentler, 1999). However, these indices are considered a guide, and not absolute values (Hu and Bentler, 1999). Gender was not controlled for as previous goal motive research found no gender differences (Ntoumanis et al., 2014; Sheldon and Elliot, 1999).

## Results

### Descriptive statistics

Data were screened for partially completed and ineligible participant responses before being screened for outliers using univariate and multivariate screening. This resulted in data for 323 participants being used for analysis (mean age = 32.46 ± 13.12 years; *n*_male_ = 135, *n*_female_ = 188; Caucasian = 240, Black = 50, Asian = 17, Other = 16; Area: Suburban = 107, Urban = 127, Rural = 85, Other = 4; Occupation: Student = 129, Office/Desk role = 96, Teacher/Educator = 39, Other = 37, Unemployed = 7, Labourer = 7, Fitness Instructor/Coach = 6, Driver = 2). The means, standard deviations, and correlations between the variables stated in the model are presented in Table 1.

**Table 1.** Model variables means, standard deviations, correlations (r).

Variable	1.	2.	3.	4.	5.	6.	7.
1. Autonomous goal motives	–	–	–	–	–	–	–
2. Controlled goal motives	−.03	–	–	–	–	–	–
3. Self-efficacy	.32**	−.17**	–	–	–	–	–
4. Motivation	.69**	−.13*	.32**	–	–	–	–
5. Affect	.64**	.00	.26**	.82**	–	–	–
6. Physical activity (minutes of MVPA)	.21**	.06	.13*	.36**	.37**	–	–
7. Mental well-being	.23**	−.17**	.59**	.28**	.25**	.09	–
*M*	5.28	2.80	31.13	9.79	4.73	264.40	22.11
*SD*	1.51	1.37	4.56	7.10	1.40	311.38	3.53

*Note. N* = 323.

* *p* < 0.05. **p < 0.01, two-tailed; MVPA: Moderate-Vigorous Physical Activity.

As shown in Table 1, autonomous goal motives were significantly associated with higher reported self-efficacy, greater quality of motivation, greater positive affective experiences, higher levels of reported MVPA, and greater perceived mental well-being. In contrast, controlled goal motives were found to be significantly associated with lower self-efficacy, poorer-quality motivation, and poorer perceived mental well-being.

### Structural equation model path analysis

**Figure 2.**
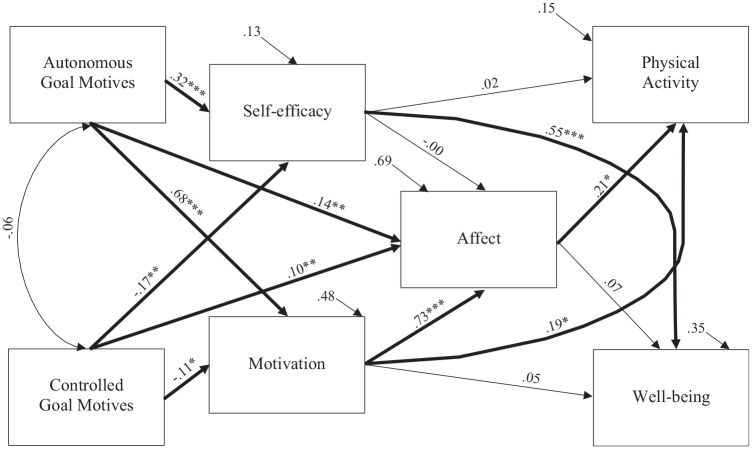
Model showing the associations between goal motives, psychological variables, physical activity and well-being. Note.*p < .05. **p < .01. ***p < .001.

The data demonstrated good fit to the proposed model: χ^2^ (6) = 14.162, *p* = 0.028, RMSEA = 0.065, CFI = 0.990, TLI = 0.965 (Hu and Bentler, 1999). Direct and indirect associations of the proposed model are presented in Figure 2.

#### Goal motives

##### Autonomous goal motives

(H1) Autonomous goal motives had a significant, positive, small-to-large association with greater self-efficacy (β = 0.32, 95% CI [0.21, 0.42], *p* = 0.001), positive affective experiences (β = 0.14, 95% CI [0.05, 0.24], *p* = 0.003), and quality of motivation (β = 0.68, 95% CI [0.61, 0.74], *p* = 0.001). (H3) Autonomous goal motives also showed small significant, indirect, positive associations with greater perceived mental well-being, via self-efficacy (β = 0.41, 95% CI [0.26, 0.58], *p* = 0.001) and higher reported MVPA via motivation (β = 26.27, 95% CI [3.84, 52.58], *p* = 0.02), affect (β = 6.28, 95% CI [0.95, 15.34], *p* = 0.02), and through motivation *and* affect (β = 21.60, 95% CI [1.56, 41.52], *p* = 0.04).

##### Controlled goal motives

(H1) When goals were underpinned by controlled motives, the model showed a significant, direct association with lower reported self-efficacy (β = −0.17, 95% CI [−0.27, −0.06] *p* = 0.004) and poorer quality of motivation (β = −0.11, 95% CI [−0.20, −0.02] *p* = 0.02), both with small effect sizes. Controlled motives had a significant, small positive association with positive affective experiences (β = 0.10, 95% CI [0.03, 0.16] *p* = 0.002). (H3) Indirectly, the pursuit of goals with controlled motives was found to have small significant associations with poorer perceived mental well-being through self-efficacy (β = −0.23, 95% CI [−0.40, −0.08], *p* = 0.003). Controlled motives were also reportedly, indirectly associated with lower minutes of MVPA via motivation (β = –4.51, 95% CI [−12.44, −0.60], *p* = 0.02), and motivation *and* affect (β = −3.71, 95% CI [−10.19, −0.28], *p* = .03); but were indirectly associated with higher MVPA via affect (β = 4.68, 95% CI [0.45, 11.48], *p* = 0.03).

#### Psychological variables

##### Self-efficacy

(H2) In the final model, perceived self-efficacy was significantly, largely, and directly associated with greater perceived mental well-being (β = 0.55, 95% CI [0.47, 0.62] *p* = 0.001). Conversely, self-efficacy was not directly associated with greater positive affective experiences (β = –0.00, 95% CI [–.07, 0.08] *p* = 0.99), nor was it directly associated with reported MVPA (β = 0.02, 95% CI [–0.10, 0.14] *p* = 0.75). Additionally, self-efficacy was not found to be indirectly associated, via affect, with reported MVPA (β = –0.02, 95% CI [–1.15, 1.26], *p* = .92) or perceived mental well-being (β = 0.00, 95% CI [–0.01, 0.01], *p* = .084).

##### Motivation

(H2) The quality of one’s motivation was directly and significantly, largely associated with greater positive affective experiences (β = 0.73, 95% CI [0.65, 0.80] *p* = 0.001) and showed small associations with higher reported MVPA (β = 0.19, 95% CI [0.02, 0.36] *p* = 0.03), yet was not directly associated with perceived mental well-being (β = 0.05, 95% CI [–0.11, 0.21] *p* = 0.51). Furthermore, quality of motivation was significantly, associated, through affect, with higher reported MVPA indirectly (β = 6.74, 95% CI [0.31, 12.64], *p* = 0.04), but not indirectly associated with perceived mental well-being (β = 0.02, 95% CI [–0.03, 0.08], *p* = 0.40).

##### Affect

(H2) Greater positive affective experiences appeared in the model to have a small direct significant associated with greater reported MVPA (β = 0.21, 95% CI [0.02, 0.39] *p* = 0.03), but affective experiences were not directly associated with perceived mental well-being (β = 0.07, 95% CI [−0.08, 0.23] *p* = 0.39).

## Discussion

This study aimed to enhance understanding of the association between goal motives and psychological outcomes, physical activity, and mental well-being by answering the following questions: (1) are psychological variables associated with improved physical activity and well-being linked to one’s motives for pursuing physical activity goals?; (2) how do psychological variables link to physical activity and well-being?; (3) what are the indirect effects of one’s motives for physical activity on physical activity and well-being? Overall, our findings supported our hypotheses. First, H1 was accepted as both autonomous and controlled motives were significantly associated, positively or negatively respectively, with all psychological variables. Second, H2 was partially accepted as significant associations were observed between both motivation and affect and physical activity, and between self-efficacy and mental well-being but not between all three psychological variables and physical activity and mental well-being. Lastly, H3 was partially accepted as significant indirect associations were found between autonomous motives and physical activity and mental well-being, and controlled motives and mental well-being, but not between controlled motives and physical activity. The findings of the present study emphasize the importance of measuring physical activity and well-being simultaneously when assessing long-term adherence.

Considering autonomous and controlled motives are described broadly in SDT as the motives for behavior (Deci and Ryan, 1985) and that SDT proposes the continuum of motivation types that are then categorized into the two broad motives (Ryan and Deci, 2017), the finding that controlled motives and quality of motivation were not significantly associated with each other was in line with these propositions. Further, more self-regulated motivation, which can lead to an increased likelihood of adoption and maintenance of new behaviors (Teixeira et al., 2012, was positively associated with increased belief in one’s ability to complete the desired behavior. Blom et al. (2021) suggested that although it takes time to change physical activity behaviors, initially improving autonomous motives and self-efficacy could be beneficial and are suggested to be the first stages in changing long-term behaviors. These findings also suggest that when an individual’s autonomy for the activity is greater, they should have higher quality of motivation (i.e. intrinsic and identified motivation; Ryan and Deci, 2017) and positive affective experiences, specifically attraction to the activity. As a result, being attracted to activities that increase moderate-vigorous activity could lead to increased adoption of healthy physical activity behaviors (Ekkekakis and Brand, 2019). This idea is supported by both the direct and indirect association between motivation and physical activity in this study.

As hypothesized (H1), controlled motives were associated with significantly lower self-efficacy and quality of motivation. If controlled motives result in lower confidence in one’s ability to complete a goal/be more active, pursuing goals with higher controlled motives could have adverse effects on physical activity adoption and maintenance; as lower self-efficacy can influence the time and effort invested to achieve a goal, thus reducing the likelihood of goal attainment (Bandura, 2001). In addition, lower RAI scores correspond with more introjected and extrinsic motivation (Ryan and Deci, 2017) or, more simply, controlled motives. Thus, our findings further demonstrate the negative associations between these controlled goal motives, motivation and self-efficacy, the latter two of which have been linked to poor long-term physical activity adherence. In contrast to self-efficacy and motivation, controlled motives were positively associated with positive affective experiences (Sheldon and Elliot, 1998). When physical activity is perceived to be pleasurable, individuals are more likely to maintain it (Schmid and Reimann, 2019). However, given the findings of this data report a snapshot in time, and as controlled motives are not considered to be enduring over an extended period (Emm-Collinson et al., 2020), these relationships may prove different over time; the temporal nature of these relationships require further investigation in future research.

Although hypothesized that all psychological variables would be positively associated with physical activity and mental well-being (H2), this was not found to be the case. Self-efficacy has been suggested to be the best mediator of physical activity (Bauman et al., 2012; Sallis et al., 1989), yet the present study found no significant association between the two variables in this population. However, a significant positive association was found between one’s belief and confidence in their abilities to complete the task and perceived well-being. As well-being and physical activity are essential variables for consideration when addressing long-term behavior change (Rhodes and Kates 2015; Schmid and Reimann, 2019), these findings emphasize the need to assess psychological outcomes and physical activity simultaneously when evaluating the effects of physical activity interventions, something that is currently lacking (Garstang et al., 2024). Previously, however, positive experiences have been shown to be more important than self-efficacy at predicting physical activity behaviors (Lewis et al., 2016). Consequently, the current study supports those previous findings as alongside quality of motivation, positive affective experiences were positively associated with higher levels of physical activity. The findings of the current study thus reinforce the importance of considering multiple psychological variables in the pursuit of positive health behaviors and the role each may have in long-term behavior change.

In line with the goal motives literature (Deci and Ryan, 2008; Emm-Collinson et al., 2020; Maltby and Day, 2001; Sheldon and Elliot, 1998), higher autonomous motives for goal pursuit were significantly associated with greater perceived mental well-being and higher levels of physical activity. In contrast, in this study, we found controlled motives were significantly and indirectly related to poorer mental well-being, which is somewhat consistent with past research that found controlled motives were directly related to poorer mental well-being (Briki, 2016; Maltby and Day, 2001; Ng et al., 2012). Previous findings in relation to exercise also support this (e.g. Standage et al., 2008), by suggesting that controlled motives are not associated with physical activity. As such, these current findings provide further evidence to support promoting autonomous motives and limiting controlled motives for physical activity goals, as they do not fulfil one’s psychological needs (Hagger et al., 2014). Furthermore, as the association between physical activity and well-being is bidirectional, perceived mental well-being and psychological variables (e.g. self-efficacy and motivation) can impact upon maintained physical activity behaviors (Kim et al., 2020) and should be considered in future studies.

### Implications

Based on the findings of this study, we suggest a number of implications. First, autonomous motives offered greater benefits to psychological variables associated with repeated and sustained engagement in physical activity, and well-being compared to controlled motives. Therefore, individuals, researchers and practitioners should seek to underpin future goal pursuits with autonomous motives to avoid potential detrimental effects that could lead to disengagement, and in the case of physical activity, sustained inactivity. Further, it is important for future goal-setting research to consider goal motives for goal pursuit as one’s quality of motivation could have significant impacts on physical activity behavior in the long-term, as often the focus is only on the type of goal set. Second, and relatedly, the findings underscore the importance of considering motives in the process of goal setting (e.g. Bird et al., 2024). Consequently, we suggest that guidance surrounding goal setting for physical activity should emphasize the importance of understanding the motives that underpin goal pursuit and thus go beyond solely focusing on the content of a goal (e.g. how specific, measurable, or challenging is it?). Third, our findings demonstrate the importance of considering psychological outcomes that can contribute to the outcomes of physical activity behavior rather than solely focusing on physical activity alone. Consideration of these psychological factors is important when seeking to understand goal setting and physical activity in future as this will allow for a more holistic approach to setting goals with different motives.

### Limitations and future directions

This study is the first to offer insight into the association between goal motives, psychological variables influencing, and the outcomes of, physical activity and well-being; yet is not without limitation. Firstly, data reported in this study are cross-sectional and represent a single time point, and it should be noted, although physical activity was not restricted by COVID-19 during the data collection period, the pandemic did alter attitudes, intentions and behaviors. Subsequently, causality cannot be inferred nor firm conclusions about the mechanisms between these variables offered. Nevertheless, we still, offer initial insight into the associations between key psychological variables in relation to the pursuit of physical activity goals. We also note that the high correlation between affect and motivation should be considered when interpreting these results as any changes could be a result of the effect have on the other. Future research may aim to examine these variables using a longitudinal approach, with objective measures of physical activity, to gain a better understanding of their interactions over time. Secondly, this study recruited a UK sample, therefore potentially limiting the applicability of these findings globally. Future research may look to recruit individuals from multiple countries to account for any geographical and cultural differences. Furthermore, few studies have sought to integrate concepts from goal motives (e.g. SCM; Sheldon and Elliot, 1998) and goal setting (e.g. goal-setting theory; Locke and Latham, 2002). Future research may look to explore this, which could better our current understanding of health behaviors.

### Conclusion

The present study offers insight into the intricacies of how goal motives are associated with psychological variables linked to improved physical activity and well-being, in turn illustrating the importance of measuring physical activity and well-being simultaneously when assessing long-term adherence. Autonomous motives were found to be associated with higher levels of physical activity and greater well-being, whereas controlled motives were associated with poorer well-being, suggesting that the promotion of autonomous goal motives would be most advantageous for health behaviors. To summarize, goal motives were associated with psychological variables linked to physical activity and well-being, with the proposed model indicating that the relationship between goal motives, physical activity, and well-being was not direct, but was influenced by perceived self-efficacy, motivation, and affective experiences.
